# Precious Cargo: The Role of Polymeric Nanoparticles in the Delivery of Covalent Drugs

**DOI:** 10.3390/molecules29204949

**Published:** 2024-10-19

**Authors:** Daniel Weissberger, Martina H. Stenzel, Luke Hunter

**Affiliations:** School of Chemistry, The University of New South Wales (UNSW), Sydney, NSW 2052, Australia

**Keywords:** targeted covalent inhibitors, reactive drugs, electrophilic warheads, drug delivery, nanomedicine, polymeric nanoparticles, pharmacodynamics, bioavailability, drug metabolism, cancer treatment

## Abstract

Covalent drugs can offer significant advantages over non-covalent drugs in terms of pharmacodynamics (i.e., target-binding properties). However, the development of covalent drugs is sometimes hampered by pharmacokinetic limitations (e.g., low bioavailability, rapid metabolism and toxicity due to off-target binding). Polymeric nanoparticles offer a potential solution to these limitations. Delivering covalent drugs via polymeric nanoparticles provides myriad benefits in terms of drug solubility, permeability, lifetime, selectivity, controlled release and the opportunity for synergistic administration alongside other drugs. In this short review, we examine each of these benefits in turn, illustrated through multiple case studies.

## 1. Introduction

### 1.1. History of Covalent Drugs

**Figure 1:**
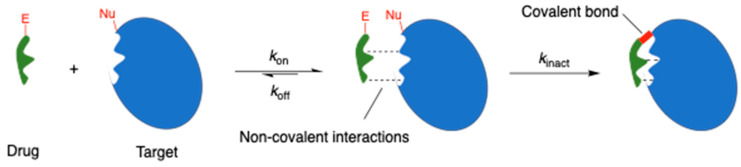
Mechanism of binding of a covalent drug to its biological target. E = electrophilic “warhead”; Nu = nucleophile.

Covalent drugs contain a reactive functional group, or “warhead”, that can form a strong chemical bond with the biological target (Figure 1) [1]. This definition includes prodrugs that are metabolised inside the body to produce reactive species in their active form. The warheads of covalent drugs are usually electrophilic in nature, ranging from mildly reactive (e.g., acrylamides, aziridines, esters, nitriles) to highly reactive (e.g., chloroethylamines, nitrogen mustards, epoxides). This electrophilic reactivity is complementary to the nucleophilic functional groups commonly found within biological macromolecules, such as the cysteine residues of proteins or the nitrogen atoms of DNA bases.

The simple act of forming a covalent bond between a drug and its target has a significant effect on the drug’s pharmacodynamic properties. Permanent blockage of the binding site usually forces the target to undergo resynthesis before its activity can be re-established, leading to a longer therapeutic effect and improved potency of the drug [2,3]. Covalent drugs can be advantageous for treating diseases in which high target occupancy is important, such as cancer and bacterial infections [2,3]. It may be possible to administer covalent drugs at lower, less frequent doses, which can reduce toxicity and improve patient comfort and compliance. Finally, covalent drugs can successfully address what would otherwise be considered “undruggable targets”, i.e., intractable proteins that have shallow binding pockets where reversible drugs cannot bind [4].

**Figure 2:**
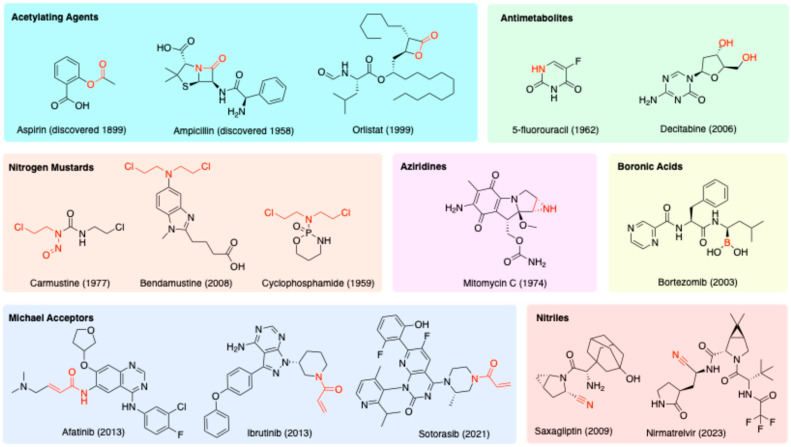
Common reactive moieties (highlighted in red) seen in covalent drugs; the year in brackets specifies the date of discovery or FDA approval.

Covalent drugs have a long history in the pharmaceutical industry, stretching back to the discovery of aspirin in 1899 for the treatment of pain and inflammation (Figure 2). Aspirin remains the most widely used medication today [4], and covalent drugs now account for approximately 7% of all small-molecule drugs approved by the Food and Drug Administration (FDA) [5]. Numerous review articles have highlighted the sustained interest in designing novel covalent drugs over recent decades [2,3,4,5,6,7,8,9,10,11,12].

Many historical covalent drugs were discovered without any knowledge of their mechanism of action. In the case of aspirin (Figure 2), it was found only much later that the therapeutic effect is attributable to the inhibition of the enzyme cyclooxygenase [13]. The ester moiety of aspirin acts as an acyl transfer reagent, which irreversibly acetylates Ser530 of the enzyme. Another type of acylating drug is the β-lactam class of antibiotics, e.g., ampicillin (Figure 2). The ring strain of the lactam (a cyclic amide), compounded by the presence of a fused ring, forces the nitrogen into a trigonal pyramidal geometry. This makes the adjacent carbonyl more electrophilic and prone to ring-opening by nucleophiles [14]. β-Lactam antibiotics inhibit important enzymes responsible for building cell walls in both Gram-positive and Gram-negative bacteria [15]. The lactone (cyclic ester) variant is present in the drug orlistat (Figure 2). Orlistat is used to treat obesity by inhibiting fatty acid synthase, but it has been recently investigated for the treatment of cancer, as fatty acid synthase is often overexpressed in cancer.

Some drugs, such as 5-fluorouracil and decitabine (Figure 2), can harness enzymes to form covalent bonds with DNA. Such drugs are known as antimetabolites; they are structural analogues of purines and pyrimidines and can thus act as atypical DNA building blocks. The generation of aberrant/damaged DNA makes these drugs useful in chemotherapy to kill rapidly dividing tumour cells [16].

Irreversible DNA binding is further exploited with the reactive nitrogen mustards (Figure 2). Nitrogen mustards contain the bis(2-chloroethyl)amino functional group, which spontaneously expels chloride to form an aziridinium intermediate that can alkylate the nucleophilic sites on DNA bases [17]. Repetition of this process with the second chloroethyl group of the nitrogen mustard allows a second covalent bond to be formed with DNA, leading to crosslinks which prevent DNA replication and ultimately result in the apoptosis of the cell [18]. A prominent nitrogen mustard, cyclophosphamide, was developed in the 1950s. Bendamustine was discovered soon after in East Germany but was not approved by the FDA until half a century later in 2008 [19]. Carmustine, approved in 1977, is a related structure. Despite the known toxicity of these compounds, they are still considered acceptable in chemotherapy due to the gravity of cancer as a disease.

Functionally like the nitrogen mustards are the aziridines, e.g., mitomycin C (Figure 2). Aziridines become activated by protonation, and the resulting aziridinium resembles the activated intermediate derived from nitrogen mustards. However, aziridines are subtly less reactive than mustards, because the charge of the protonated aziridinium is somewhat dissipated by solvation. Therefore, aziridines are more stable and less likely to be inactivated by off-target nucleophiles like water and glutathione.

Reversible covalent bonding groups, which strike a balance between the benefits of non-covalent and covalent drugs, have also been used [20]. The boron-containing bortezomib (Figure 2) is a proteasome inhibitor designed to treat multiple myeloma. The boron reacts with a threonine hydroxyl group on the 20S proteasome to form a boronate [6].

Michael acceptors (Figure 2) are another important category of electrophilic warheads, typically targeting cysteine residues within protein binding sites [21,22]. Exemplifying this category are the drugs ibrutinib and afatinib, which are tyrosine kinase inhibitors, and sotorasib, which is a guanosine triphosphatase (GTPase) inhibitor. During the development of these drugs, there was a strong emphasis on optimising the non-covalent binding interactions, to maximise selectivity for the desired target over off-targets. Drugs that emerge from such an approach are sometimes referred to as targeted covalent inhibitors (TCIs).

The final category of electrophilic warhead depicted in Figure 2 is the nitrile, as seen in the drugs saxagliptin and nirmatrelvir. Saxagliptin is a dipeptidyl peptidase-4 (DPP-4) inhibitor and anti-diabetic and has potential to treat Alzheimer’s disease [23]. Nirmatrelvir, which is an antiviral drug that targets the main protease of severe acute respiratory syndrome coronavirus 2 (SARS-CoV-2), was discovered by an electrophile-first approach: instead of building from a known reversible inhibitor, an electrophile was chosen, and the rest of the structure was expanded from it [6].

### 1.2. Disadvantages of Covalent Drugs

The primary disadvantage of covalent drugs is their potential to form irreversible bonds with off-target proteins, which can lead to unpredictable downstream effects [24]. In some cases, unexpected drug–protein adducts can induce idiosyncratic immune responses that are harmful to patients [25,26]. The negative consequence of off-target binding is compounded by the fact that less drug will reach the desired target. As was discussed above with TCIs, it is possible to impart some selectivity for the desired target by optimising the non-covalent interactions, but the issue of off-target binding remains a concern.

Another disadvantage of covalent drugs is their susceptibility to metabolism. Due to their reactive nature, covalent drugs can be easily degraded and inactivated. For example, increased expression of glutathione is a significant factor in cancer drug resistance: partly due to this, the nitrogen mustards bendamustine and carmustine both have short half-lives of around 30 minutes [17]. Meanwhile, afatinib suffers from significant extrahepatic metabolism by reactivity with glutathione [27].

For a time, these disadvantages caused the development of covalent drugs to be seen as a risky endeavour. During the advent of high-throughput screening of drug candidates in the 1980s, compounds that covalently bind to proteins were generally excluded from compound libraries due to fears that they could bind to random proteins and cause toxicity [28,29]. The overall hesitancy of the pharmaceutical industry to invest in covalent drug research means that covalent drugs may have yet to reach their full potential [2,4,30].

### 1.3. Nanoparticles as a Possible Solution

Drug delivery systems are a useful way of mitigating some of the problems of drugs by protecting them until they are released at their destination in a controlled, sustained manner. Research has progressed from conventional delivery systems such as tablets and capsules to controlled-release hydrogels and matrices and recently to more advanced technologies like nanomedicine [31]. Nanoparticles are useful in that they are able to carry a payload of drugs while being small enough to cross biological barriers, be distributed locally and avoid embolisms [32].

**Figure 3:**
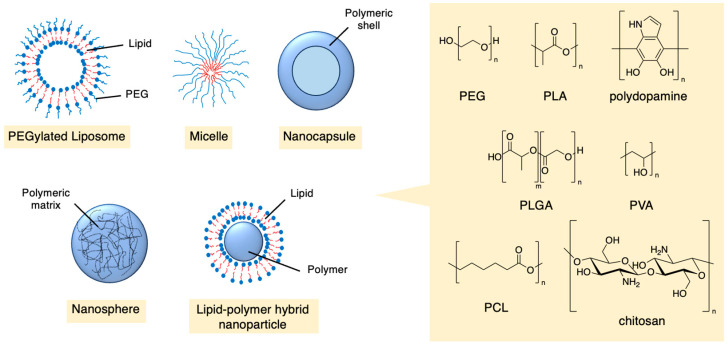
Architectures and chemical structures of some polymeric nanoparticles that have been used in drug delivery.

Nanoparticles can be constructed from a range of materials, such as lipids, polymers, carbohydrates, proteins, inorganic substances and metal-organic frameworks (MOFs) [33,34]. They can form various structures like liposomes, micelles, dendrimers and worm-like particles and can easily be modified to be imaged in vitro and in vivo. Polymers have been widely used in the development of drug delivery systems, owing to their ability to self-assemble into many sizes and shapes (Figure 3). Many polymers are biocompatible, meaning they are non-toxic, are metabolised or hydrolysed into non-toxic compounds and can be efficiently expelled from the body once they release their payload. Many of these materials can act as treatments themselves, potentially bypassing multi-drug resistance [35]. Commonly used polymers include polyethylene glycol (PEG), polylactic acid (PLA), polydopamine, poly(lactic-co-glycolic acid) (PLGA), polyvinyl alcohol (PVA), polycaprolactone (PCL) and chitosan.

It is important that the drug and material used to formulate the nanoparticles are compatible. Strong van der Waals and hydrogen bonding interactions between the two can increase the drug loading capacity and delay the rate of release. With respect to covalent drugs, the warheads need to be compatible with any potentially reactive moieties within the nanoparticle. Finally, drugs can be conjugated to nanoparticles, and so appropriate linker groups need to be considered to connect the two entities together. For example, the carboxylic acid side chains of bendamustine allow for easy conjugation to polymers [36].

**Figure 4:**
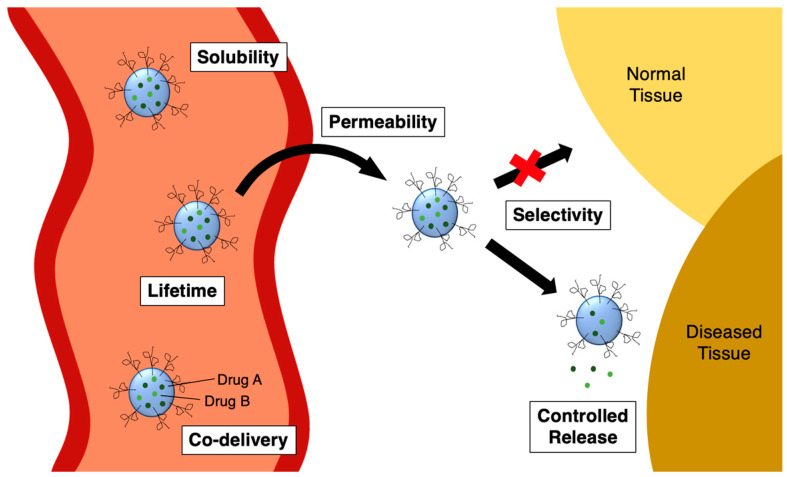
Benefits of nanoparticles for drug delivery.

Nanoparticles provide several key benefits to drug delivery. First, they can improve the solubility of hydrophobic drugs (Figure 4, “solubility”). This is because the interiors of the nanoparticles are usually hydrophobic in nature, too. Second, they can enhance a drug’s ability to cross biological membranes such as the intestine and the blood–brain barrier (BBB) (Figure 4, “permeability”). This can be achieved in conditions across a range of pH values. Third, the half-life of drugs can be extended by preventing metabolism and inactivation of the covalent warheads, allowing more circulation time within the body (Figure 4, “lifetime”). Fourth, the rate at which the drug reaches its target can be fine-tuned by the composition of the nanoparticle, which can further prolong the therapeutic effect while reducing side effects (Figure 4, “controlled release”). These four benefits can be considered together under the umbrella idea of bioavailability.

Further advantages are offered besides bioavailability. A fifth benefit is that nanoparticles can prevent the non-specific binding by the covalent warheads and allow tissue selectivity through active targeting, the latter of which is highly important in cancer and infectious diseases (Figure 4, “selectivity”). Finally, a sixth benefit offered by nanoparticles is the opportunity for the co-delivery of drugs (Figure 4, “co-delivery”). Each of these benefits will be examined in detail in Section 2 of this review.

### 1.4. Scope of This Review

A plethora of review articles have covered the drug delivery literature [31,33,37,38,39,40,41,42,43], but none of them has focused exclusively on covalent drugs. Likewise, there is a large and growing literature on covalent drugs [2,3,4,5,6,7,8,9,10,11,12], but there has not yet been a systematic review of drug delivery strategies for them. In this review, we aim to fill this gap. We chose to organise our review according to the various benefits that nanoparticles can offer for the delivery of covalent drugs. Most of these benefits apply to non-covalent drugs too, but they are especially relevant for covalent drugs.

Finally, it should be noted that a majority of the examples presented in this review relate to the treatment of cancer. This is no accident: the treatment of cancer is a dominant theme in both the covalent drug literature and the drug delivery literature. However, we have decided to take a “disease agnostic” approach in the organisation of our review, and hence, the anticancer examples will be interspersed amongst the examples that focus on other diseases.

## 2. Benefits of Nanoparticles

### 2.1. Solubility

Poor solubility is a major detractor to drug absorption and bioavailability. A typical example is seen with the drug orlistat (Figure 2). When used as an anti-obesity drug, the site of action of this drug is within the digestive tract, and hence, the drug needs to reach that location, yet it has very low aqueous solubility [44]. The typical way that this problem is tackled is to formulate orlistat with the surfactant sodium dodecyl sulfate, but this surfactant is, unfortunately, a minor stomach irritant [45]. Compounding the difficulty of administering orlistat is that much of the drug is lost during first-pass metabolism, meaning that high, frequent doses need to be administered in order to achieve the desired effect, with the undesired consequences of more side effects. The problem of poor aqueous solubility also poses considerable difficulties when orlistat is used as an anticancer drug.

**Figure 5:**
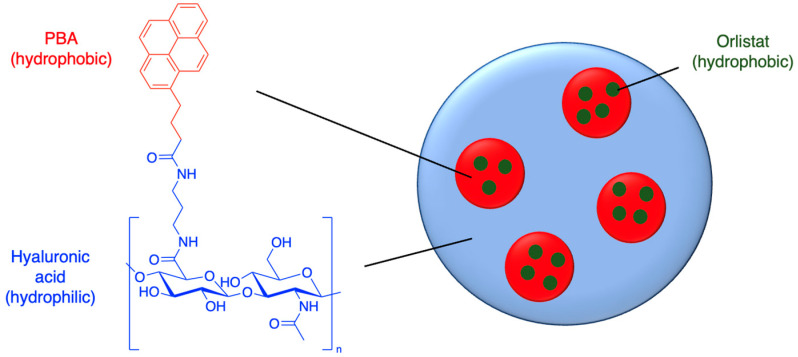
Orlistat encapsulated into the hydrophobic domains of PBA-hyaluronic acid nanoparticles (idealised, based on Hill et al. (2016)); also see Table 1, entry 1 [46].

Nanoparticles offer a potentially superior method for the delivery of orlistat. Hill et al. (2016) synthesised hyaluronic nanoparticles conjugated with the hydrophobic molecule aminopropyl-1-pyrenebutanamide (PBA) (Figure 5 and Table 1, entry 1) [46]. This nanoparticle contains hydrophobic domains where orlistat can reside. Almost all of the drug was able to be encapsulated (97% encapsulation efficiency [EE]), and the optimised nanoparticles had an impressive drug loading capacity ([LC], i.e., 19% of the mass of the loaded nanoparticle was the drug). Hyaluronic nanoparticles are generally known to be selective to cancer cells. In this case, the nanoparticles had relatively large diameters of up to 600 nm, which could affect their biodistribution; nevertheless, cell viability studies against prostate and breast cancer cell lines showed that the orlistat-loaded nanoparticles were not only more cytotoxic, but their cytotoxicity also did not diminish after preincubation in serum-free culture medium, in contrast to the free drug [46].

Several other nanoparticle systems have been developed to enhance the solubility of covalent drugs.

Hyaluronic acid, PLGA and lipids have been combined to form nanoparticles that are capable of co-encapsulating orlistat and another drug (Table 1, entry 2) [47]. A high orlistat encapsulation efficiency was achieved (90%), and the presence of the hyaluronic acid slowed drug release. The same study also performed a mice xenograft model experiment: the nanoparticles were able to be injected and significantly accumulated at the tumour site and displayed minimal systemic toxicity [47].

Another approach to solubilise orlistat is the emulsion–diffusion–evaporation technique, with the intention of treating triple-negative breast cancer. Bhargava-Shah et al. (2016) developed orlistat-loaded PLGA-PEG nanoparticles, via emulsion of ethyl acetate and 2% polyvinyl alcohol (Table 1, entry 3) [48]. The emulsion–diffusion–evaporation technique gave smaller nanoparticles with a lower polydispersity index compared to nanoparticles prepared by nanoprecipitation. Treatment against MDA-MB231 and SKBR3 cells induced apoptosis and showed a greater decrease in cell viability compared to free orlistat [48].

In another study, orlistat was loaded into self-assembling polydopamine, where an emulsion of drug-containing octane and aqueous sodium hydroxide allowed the polymer to form hollow capsules around the octane droplets (Table 1, entry 4) [49]. Polydopamine adds synergistic benefits, since the auto-oxidation of the dopamine monomers can lead to reactive oxygen species (ROS) that are harmful to cancer cells. Although the orlistat drug loading was not determined, the encapsulation efficiency of Nile red (which has similar solubility properties to orlistat) was found to be 91%. Furthermore, while the insoluble free orlistat suspension aggregated, the orlistat-loaded hollow capsules were well dispersed in water. The encapsulated drug had a greater cellular uptake and reduced cell viability against MCF7 and MDA-MB-231 cell lines [49].

Another drug that suffers from poor solubility, especially at high pH, is ibrutinib (Figure 2). Research has focused on using nanoparticles to improve the solubility of this drug for intravenous administration. For example, Rangaraj et al. (2019) developed an ibrutinib nanosuspension stabilized by the triblock copolymer, Pluronic F-127, which increased the solubility of the drug 21-fold (Table 1, entry 5) [50]. The nanosuspension had a higher drug release compared to the free drug from the fasted-state simulated intestinal fluid, and the variability compared to the non-fasted state was minimised [50].

Pluronic F-127 has been further used to stabilise PLGA nanoparticles. Ibrutinib-loaded PLGA nanoparticles, when administered orally to Wistar albino rats, had a four-fold higher absorption and bioavailability, indicating improved solubility (Table 1, entry 6) [51].

Zhao et al. (2020) incorporated ibrutinib into sulfobutylether-β-cyclodextrin (SBE-β-CD), which was then encapsulated into chitosan nanoparticles (Table 1, entry 7) [52]. Higher concentrations of SBE-β-CD led to increased water solubility and encapsulation efficiency of ibrutinib. The relationship between drug solubility and SBE-β-CD concentration was linear, with a maximum recorded solubility of 1.28 mM [52].

### 2.2. Permeability

Drugs may need to cross several biological barriers before reaching their target, depending upon their route of administration. Most drugs are administered orally, and so a major challenge for these drugs is absorption via the gastrointestinal (GI) tract, which can result in a large portion of the drug not even entering the bloodstream. For covalent drugs, this has the potential to lead to off-target effects. Furthermore, drugs passing through this route are susceptible to first-pass metabolism and are rapidly eliminated from the body. Alternative routes of administration such as the transdermal, ocular and inhalable routes bypass the GI tract but need to traverse other barriers of their own. A second barrier for drugs that target the brain is the BBB. These problems can be solved by designing nanoparticles to engage in receptor-mediated transcytosis pathways. Finally, drugs with intracellular targets need to pass the cell membrane, which hydrophilic drugs may have difficulty with.

**Figure 6:**
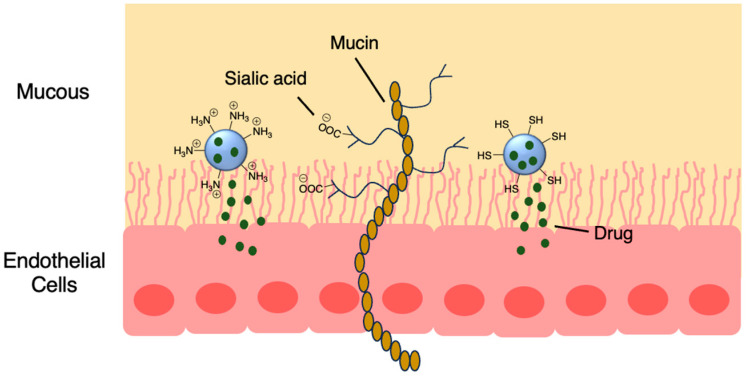
Mucoadhesive particles are able to bind to proteins within the mucous layer via disulfide and electrostatic interactions while unloading the drug payload; also see Table 2, entry 1 [57].

Nanoparticles made from chitosan [53], PLGA [54] and polyalkylcyanoacrylate [55] have garnered interest due to their permeable and mucoadhesive properties. Mucous membranes consist of a layer of epithelial cells covered by mucous secretions (Figure 6). Interactions between nanoparticles and mucus membranes are important because the nanoparticle must penetrate the mucus fast enough before it is washed away. Mucin proteins within the mucus are negatively charged due to sialic acid and ester sulfate groups on the carbohydrate branches, but there are also areas of hydrophobicity. Therefore, nanoparticles with positively charged groups and hydrophobic surfaces typically have mucoadhesive properties. Thiol groups also increase mucoadhesiveness and permeation, with their ability to form disulfide bonds [56]. For example, pH-sensitive thiolated chitosan/poly(malic acid) (PMLA) nanoparticles were developed to deliver the β-lactam amoxicillin through the stomach mucous layer to treat *Helicobacter pylori* infection (Figure 6 and Table 2, entry 1) [57].

Using an alternative route of administration for drugs can allow better patient compliance, as well as increased selectivity when administered locally. The ocular, intranasal, inhalable and transdermal routes have all been considered for nanoparticle drug delivery.

Chitosan-pluronic nanogels transported 5-fluorouracil across the skin for the treatment of melanoma (Table 2, entry 2) [58]. In a mouse model where the nanoparticles were applied to the skin, there was minimal skin irritation and no edema formation. The nanoparticles were pH-responsive and biodegradable and allowed the drug to regenerate the squamous skin layer. The anticancer effect of a low dose was significantly higher than a high dose of the free drug [58].

Salgueiro et al. (2004) administered the nitrogen mustard cyclophosphamide as eye drops via polyalkylcyanoacrylate nanospheres to act as an immunosuppressant (Table 2, entry 3) [55]. The administration of the formulation on rabbits was well tolerated, with no corneal or conjunctival irritation. The ocular tolerance was reported as being superior to a previous study involving liposomes as the drug carrier [55].

Concerning the inhalable route, Elbatonony et al. (2021) used ultra-probe sonication to encapsulate afatinib in PLGA nanoparticles (Table 2, entry 4) [59], while Vanza et al. (2023) used a two-step double emulsion solvent evaporation (w/o/w) method (Table 2, entry 5) [60]. The latter further optimised the w/o/w method with a three-level factorial design and saw an improvement in encapsulation efficiency over the method described by Elbatonony et al. (2021) from 34% to 78%. Both formulations were converted to a dry powder inhaler form and had fine particle fractions above 60%, showing that the majority of the nanoparticles were small enough to penetrate deep into the lungs.

Targeting ligands can aid nanoparticles with crossing the BBB, as there are many receptors along the BBB that induce transcytosis. Carmustine was incorporated into solid lipid nanoparticles conjugated with tamoxifen and lactoferrin, a glycoprotein known to cross the BBB (Table 2, entry 6) [61]. The BBB was modelled using a synthetic membrane cultured with human brain microvascular cells (HMBECs). The lactoferrin caused a slight decrease in the transendothelial electrical resistance and an increase in the permeability coefficient. Although the presence of tamoxifen and lactoferrin resulted in slight toxicity to HMBECs, there was a much greater toxicity to malignant U87MG cells [61].

Fernandes et al. (2018) added valine to saxagliptin-loaded chitosan NPs to allow passage through the BBB via the large amino acid transporter (LAT-1) (Table 2, entry 7) [23]. A dye loaded into the NPs was found to localise in the brain at 65 ng/g of the tissue, whereas the free dye was directed towards mainly the liver and kidneys; furthermore, saxagliptin was detected in the brain at a concentration of 53 ng/mL after 24 h when loaded into NPs, while no detectable concentration reached the brain when administered as the free drug [23].

Lo et al. (2021) used lipid–polymer nanoparticles modified with tight junction-modulating peptides to improve afatinib transport across the BBB (Table 2, entry 8) [62]. The nanoparticles were found to cross a BBB model of bEnd.3 endothelial cells via a transcytosis pathway and by perturbing the tight junctions between the cells. The cytotoxicity of the formulation was tested on PC9 cells after permeating through the membrane, upon which there was an insignificant difference compared to an assay not involving the BBB model (~40% cell viability). This contrasted with both free afatinib and unmodified, afatinib-loaded nanoparticles, whose cytotoxicity was dampened due to the protection of the BBB model (85% and 65% cell viability, respectively) [62]. It should be noted that in this and some other examples discussed in this Section, the selectivity of the treatment for cancerous vs. normal cells was not investigated; the selectivity question will be addressed in Section 2.4.

The membrane permeability of drugs is also important for cellular uptake. One key reason why cellular uptake is necessary for covalent drugs is that cysteine residues are mainly found on intracellular proteins [63]. Almost all nanoparticles use endocytosis to pass through the negatively charged cell membrane, allowing even large drugs to be internalised. Drug efflux transporters can also be bypassed, thereby mitigating resistance in cancer [40]. Therefore, targeting multiple endocytosis pathways is advantageous in this regard [64,65]. The mechanisms of nanoparticle endocytosis are well covered in the literature [66].

Gold nanoparticles have been suggested to enter cells by non-specific-receptor-mediated endocytosis [67,68]. Afatinib was conjugated to PEGylated gold nanoparticles by coupling the afatinib amines to the terminal carboxylic acid groups on the PEG layer. The internalisation of the nanoparticles was confirmed by confocal imaging. The use of these nanoparticles led to higher cytotoxicity and lower cell growth, with IC_50_ values going from 0.50 to 0.10 μM in S2-013 cells and from 0.87 to 0.04 μM in A549 cells [68]. Hong et al. (2019) used lipid–polymer nanoparticles conjugated with pH-responsive cell-penetrating peptides to encapsulate afatinib and treat colorectal cancer. These peptides were shown to increase uptake into Caco-2 cells and afatinib cytotoxicity when in an acidic environment [69].

### 2.3. Lifetime

The half-life of a covalent drug can be significantly extended when the drug is encapsulated within a nanoparticle carrier. Direct contact with metabolic enzymes, acidic conditions, water and the immune system can be limited until the payload is released [70]. The surface properties of the nanoparticle play an important role in bioavailability. PEG is often used to coat the surface of nanoparticles, as it is a hydrophilic polymer that gives stealth-like properties. It also provides physical stability to lipid-based systems and prolongs circulation time.

**Figure 7:**
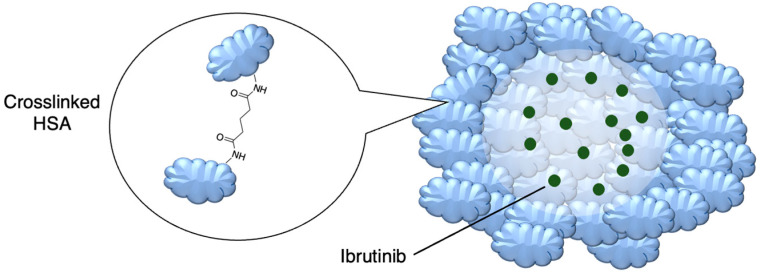
Encapsulation into crosslinked HSA nanoparticles protects ibrutinib from degradation [71]; also see Table 3, entry 1.

Prior success in using human serum albumin (HSA) nanoparticles to deliver the non-covalent drugs paclitaxel and abraxane led to this system being chosen as a candidate for delivering the covalent drug ibrutinib. Famta et al. (2023) used crosslinked HSA to load ibrutinib (Figure 7 and Table 3, entry 1) [71]. They found that an increase in crosslinker resulted in smaller particle sizes but lower drug encapsulation efficiency. The optimized nanoparticles were 124 nm with a polydispersity index of 0.113 and had an encapsulation efficiency of 90%. The half-life increased from 0.4 h to 2.9 h [71]. This system was developed further by Yang et al. (2023), who incorporated both ibrutinib and hydroxychloroquine into nanoparticles made from soybean oil and HSA. The size of the nanoparticles increased from 132 nm to 160 nm upon the inclusion of hydroxychloroquine. The nanoparticles led to six-fold higher levels of the drug at the targeted tissue than the free drug. In a mouse model, there was a higher percentage of survival compared to both the ibrutinib-only nanoparticles and free ibrutinib [72].

PEGylated delivery systems are beginning to reach the market, such as Promitil, a patented formulation of mitomycin C in PEGylated liposomes [78]. Bypassing first-phase metabolism of ibrutinib was achieved with PEGylated lipid–polymer hybrid nanoparticles comprising a PLGA core (Table 3, entry 2) [73]. Patel et al. (2023) investigated the uptake mechanism of the drug delivery system into Peyer’s patches in the intestine. They found that oral bioavailability was better, with a 23-fold increase and a doubling of the half-life. Furthermore, the amount of drug in plasma was significantly lower in rats after administering the lymphatic-flow-blocker cycloheximide, showing that the drug was being absorbed by the intestine [73].

The half-life of afatinib is mainly determined by covalent interactions with plasma proteins, rather than metabolism [79], which differentiates it from non-covalent drugs. Afatinib has been encapsulated in PEGylated liposomes to improve its pharmacokinetic properties. The liposomes were able to significantly increase the elimination half-life of afatinib by over two-fold (Table 3, entry 3) [74]. Similar results in improving the half-life were found using lipid–polymer hybrid nanoparticles (Table 3, entry 4) [75]. Loading afatinib into solid lipid nanoparticles, which were themselves placed inside of PLGA porous microspheres (Table 3, entry 5), the half-life of the drug was further extended to a time of 81 h when administered to Sprague-Dawley rats [76].

To address the limited half-life of carmustine, the drug was co-loaded with O6-benzylguanine into PLGA-chitosan core-shell nanoparticles (Table 3, entry 6) [77]. It was hypothesised that O6-benzylguanine would consume the O6-methylguanine-DNA-methyltransferase repair protein and therefore counter drug resistance. The half-life of loaded carmustine was five times longer than that of free carmustine in plasma. Rat survival rate also markedly increased upon addition of O6-benzylguanine to the nanoparticles. Meanwhile, there was no significant difference between carmustine-only nanoparticles and free carmustine solution. A further benefit of the O6-benzylguanine-loaded nanoparticle formulation was that it led to no discernable weight loss in rats during the timescale of the experiment, suggesting that the formulation is non-toxic [77].

### 2.4. Selectivity

To make covalent drugs more selective, nanoparticles can use size to discriminate between the barriers they cross. During angiogenesis in cancerous tissue, hastily grown blood vessels can be passively targeted. The endothelial walls of these blood vessels are disrupted and allow nanoparticles to leak through from the bloodstream. This observation led to researchers attempting to exploit this phenomenon using nanoparticles, which are small enough to extravasate from these blood vessels into the neighbouring tumour tissue but large enough to not penetrate through healthy, properly formed vessels. It is then possible for the nanoparticles to be retained within the tumour so that drug action can occur. This phenomenon is known as the enhanced permeability and retention (EPR) effect. Although this usually cannot be solely relied upon for selectivity in humans [42,80], it plays a role alongside active targeting approaches.

An example of the EPR effect in action was demonstrated by Guan et al. (2014) [81]. They studied the effects of afatinib-loaded PEG-PCL polymeric micelles on HER2-overexpressed tumours. The drug-loaded micelles had a hydrodynamic diameter of 160 nm and were stable at various pH over 3 days. Distribution imaging experiments in a mouse model showed that the micelles accumulated mostly at the tumour site, although there was some accumulation in the rest of the colon and the stomach [81]. After 23 days, the final tumour volume was significantly smaller compared to the tumours treated with the free drug.

**Figure 8:**
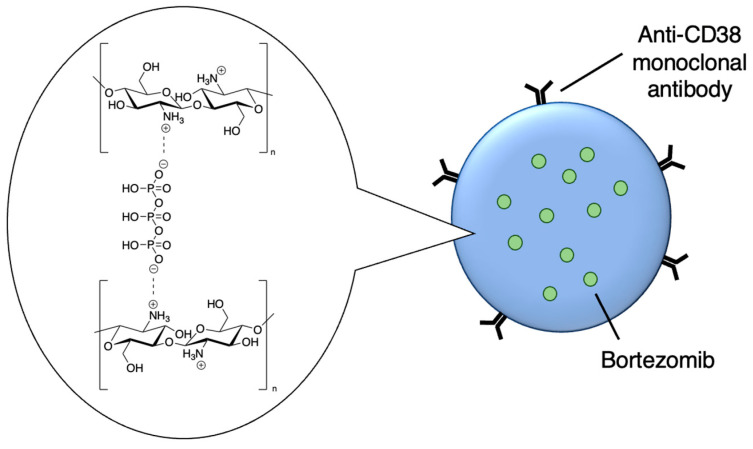
Active targeting of a bortezomib-loaded chitosan nanoparticle by attaching a CD38-targeting antibody (idealised, based on de la Puente et al. (2018)) [82]; also see Table 4, entry 1.

Drug delivery is often improved with active targeting, since the surface of the nanocarrier can be modified with antibodies or small molecule ligands to bind it to a receptor that is specific to or overexpressed in the target tissue. This allows special entry into the intended cells via endocytosis. In this way, the nanoparticles can bypass healthy tissue and minimise side effects. To this end, the CD38-targeting antibody was added onto crosslinked chitosan nanoparticles to treat multiple myeloma with bortezomib (Figure 8 and Table 4, entry 1) [82]. Although non-targeting and targeting nanoparticles had similar activity in vitro, the targeting nanoparticles performed better in vivo. This was likely due to uptake kinetics within biological systems, where non-binding particles are more easily eliminated. The authors displayed this by testing for cytotoxicity after a 2 h pulse in vitro, which resulted in a difference between the two nanoparticle types [82].

There have been several other examples of active targeting [88]. Folate-modified nanoparticles have been used to deliver 5-fluorouracil (Table 4, entry 2) [83] to a tumour. Folate, which is important for cell replication, is transported into cells via folate receptors, which are overexpressed on tumour cells [89]. In vitro cellular uptake studies by Nho et al. (2017) indicated that the folate allowed a higher accumulation and increased the potency of 5-fluorouracil-loaded PEGylated liposomes.

Li et al. (2014) showed that upon addition of folate to mitomycin C-loaded PEGylated phytosomes co-loaded with methotrexate, cellular uptake into HeLa cells was dramatically improved (Table 4, entry 3) [84]. The mitomycin C was not more potent than the free drug after 24 h of treatment but did show a significantly higher potency after 48 h. The authors attributed this to the sustained release of the drug from the nanoparticles. The folate nanoparticles also led to a lower tumour volume in vivo [84].

The transferrin receptor is another overexpressed receptor in cancer. Transferrin-coated lipid–polymer nanoparticles have been used to deliver afatinib into tumour cells (Table 4, entry 4) [75]. The nanoparticles were redox-sensitive as the transferrin was attached by a disulfide linkage, which was cleaved by the excess glutathione present. There was a higher concentration of afatinib present in tumour tissue when delivered by transferrin-coated nanoparticles than both free drug and drug-loaded nanoparticles without transferrin. After a month of treatment in vivo, the tumour volume was half that of the tumour treated with untargeted nanoparticles [75].

Alendronate, a calcium ion chelator, was used to target bone marrow for the treatment of myelodysplastic syndrome. Lipid–polymer nanoparticles loaded with the antimetabolite decitabine were appended with alendronate (Table 4, entry 5) [85]. There was a seven-fold increase in the drug from the targeting nanoparticles that accumulated in the femur, compared to non-targeting nanoparticles [85].

Zhang et al. (2019) used terpolymer–lipid hybrid nanoparticles to encapsulate mitomycin C and doxorubicin (Table 4, entry 6) [86]. These nanoparticles were targeted to both tumour cells and tumour-associated macrophages to treat breast cancer. Respectively, this was performed by incorporating the targeting peptide iRGD and polysorbate 80 (which is able to attract apolipoprotein E). Apolipoprotein E can be transported across endothelial cells and can bind to tumour-associated macrophages via low-density lipoprotein (LDL) receptors. Meanwhile, iRGD can bind to the overexpressed integrin receptors on tumour cells [86].

Hyaluronic acid is a polysaccharide that is unique in its selectivity to CD44 receptors, which are overexpressed on the surface of cancer cells. PEGylated polylysine nanoparticles were coated with hyaluronic acid to deliver afatinib to tumour cells. This resulted in higher levels of cellular uptake and reactive oxygen species compared to nanoparticles without hyaluronic acid [90]. In another study, Mei et al. (2024) first reported a drug delivery system that targets KRAS-TP53 co-mutant tumours with the novel acrylamide sotorasib (AMG510) (Table 4, entry 7) [87]. They made hyaluronic acid triphenylphosphonium (HA-TPP) nanoparticles that were able to target CD44 and mutant p53 proteins. Alkyltriphenylphosphonium groups were of interest due to their mitochondria-targeting ability as a lipophilic cation, which leads to the elimination of the p53 proteins. A peroxide-responsive linker was also incorporated to degrade the nanoparticles upon entering the high-ROS tumour cells, further improving the selectivity. Cellular uptake was remarkably improved, and apoptosis was shown to be mediated through mitochondrial damage [87].

### 2.5. Controlled Release

The drug release rate from nanoparticles plays a key role in determining how long the drug will remain loaded before reaching the target site. This is affected by how the drug is loaded into the nanoparticle (e.g., physical encapsulation or covalently bound), whether the drug resides in the nanoparticle’s core or near the surface, if the polymer chains are crosslinked or if the polymers are pH-responsive.

**Figure 9:**
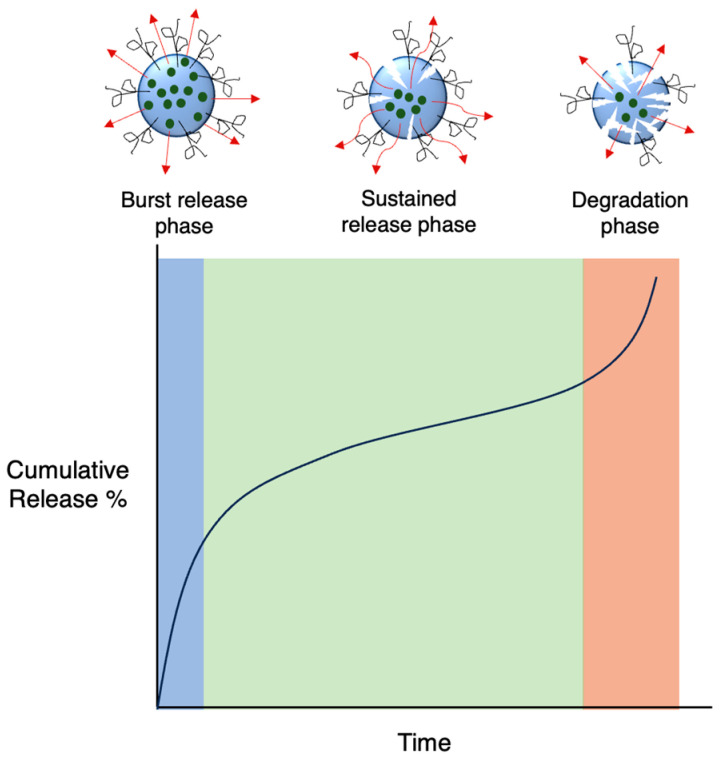
Typical representation of drug release curves of degradable nanocarriers.

In the case of biodegradable carriers with physically encapsulated drugs, drug release usually occurs in three phases (Figure 9): an initial burst release as the drug on the nanoparticle surface diffuses outwards, a much slower sustained release phase via both drug diffusion from the core and polymer degradation and a final fast release phase as the nanoparticle starts to break down completely [91]. If the nanoparticle is not degradable, only the first two phases are involved. Additionally, burst release may not always be present, in particular when there are strong forces between the drug and carrier. Although a large burst release may be sometimes preferable, minimising it is ideal in most cases as it is unpredictable and can lead to toxicity. Release kinetics can be fine-tuned based on the properties of the polymer matrix.

The biodegradable properties of commonly used polymers allow drugs to slowly be released as the polymer breaks down. One of the first marketed drug delivery systems to incorporate covalent drugs was Gliadel, a formulation of the nitrogen mustard carmustine loaded into polyanhydride-based wafers. These wafers are placed directly into the brain cavity after the excision of gliomas. As the biodegradable polyanhydride is eroded, carmustine is released in a controlled manner [92].

**Figure 10:**
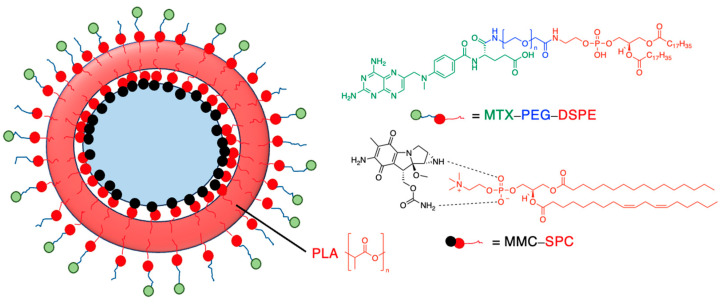
Nanoparticle system designed by Li et al. (2014); the surfactant SPC prolonged the release of mitomycin C (idealised, based on Li et al. (2014)) [95]; also see Table 5, entry 3.

Drug release can be slowed by conjugation or complexation with the nanocarrier material. For example, Hou et al. (2009) complexed mitomycin C with soybean phosphatidylcholine (SPC), which was incorporated into PLA nanoparticles via a single emulsion solvent evaporation technique (Table 5, entry 1) [93]. Although there was a slightly larger burst release compared to that from nanoparticles without SPC (likely due to the smaller size and larger surface area), the sustained release phase was prolonged. The integrity of the complex was strong enough to delay diffusion of the drug to the nanoparticle surface [93]. In a follow-up study, the same nanoparticles were prepared by a dialysis technique. In this case, the burst release of the PLA-SPC nanoparticles was reduced compared to the PLA nanoparticles, but the sustained release phase was faster, so that at the end of the experiment, the total amount of drug release was the same [94]. This suggests that the preparation method is an important factor in drug release. Finally, PEG-distearoylphosphatidylethanolamine (PEG-DSPE) was incorporated into the design to form a coating around the PLA-SPC nanoparticles (Figure 10 and Table 5, entry 3). The release profile was largely the same compared to the study by Hou et al. (2009), with the exception that a larger amount of total drug had been released at the end of the sustained release phase (~60% vs. ~45%). Although the third release phase was not observed within the timeframe of the experiments, it is desirable that any remaining drug inside the nanoparticle is minimised to avoid toxicity when the final burst release occurs. Furthermore, the use of pH-sensitive phosphatidylethanolamine within the nanoparticles allowed mitomycin C to be released faster under acidic conditions [95].

Nanoparticles can make use of biological and external stimuli to activate the release of the drug. Thermal irradiation, magnetic fields and pH changes have been used in this endeavour. External stimuli are most useful for treating diseased tissue that is close to the skin. It was found that the destruction of cells by radiosensitisation can discharge cellular components that speed up drug release. When Promitil (mitomycin C-loaded PEGylated liposomes) was in the presence of a cell culture medium, the drug was released faster when under irradiated conditions (Table 5, entry 4). This was attributed to the reducing agents that were part of the discharged cellular components [96].

Amin et al. (2023) co-loaded magnetic iron oxide nanoparticles with mitomycin C, using crosslinked PVA nanoparticles as the carrier. The magnetic properties of the iron oxide were retained, despite being impacted slightly after encapsulation [97].

5-Flurouracil-loaded nanoparticles were also modified with magnetic properties (Table 5, entry 5) [98]. Magnetite nanographene oxide polycaprolactone nanoparticles coated with chitosan guided the drug to tumour sites. Applying an alternating magnetic field to the nanoparticles slowed down tumour growth and improved the survival of colorectal-tumour-bearing mice. The magnetic field raised the temperature to 43 °C—an intentional aspect of the hyperthermia treatment—and sped up the release of the drug [98].

Gong et al. (2019) explored the use of polymeric nanovesicles to deliver afatinib for non-small-cell lung cancer (Table 5, entry 6) [99]. The nanovesicles, made from PEG-P(Asp(DBA)-co-Phe) polymers, were pH-sensitive due to the protonation of the amine groups in the polypeptide core. Little of either drug was released at pH 7.4 after 24 h, while at pH 5, the drugs experienced a burst release of up to 90% after 24 h. In vivo studies showed a smaller tumour volume and greater survival rate in rats, compared to the single-drug nanovesicles [99].

Crosslinking of the polymer matrix has also been found to impact drug release. For example, PVA contains hydroxyl side groups that can be converted into carboxylic acids. These modified groups can then be condensed with the side chains of neighbouring polymers to form crosslinks. Mitomycin C was conjugated to crosslinked PVA nanoparticles via a succinic acid linker (Table 5, entry 7) [100]. In another study, the β-lactam ampicillin was loaded into crosslinked PVA/chitosan nanofibers (Table 5, entry 8) [101]. In both cases, as the crosslinking density increased, the drug release rate slowed progressively. This was partly controlled by erosion as the ester crosslinks were hydrolysed. The slow release was also attributed to the lower surface wettability of the crosslinked nanofibers as the hydrophilic hydroxyl groups were consumed by the crosslinking process.

### 2.6. Co-Delivery of Drugs with Synergistic Abilities

Drug treatment can often be more effective when two or more drugs are administered simultaneously. This is seen especially in the case of cancer with combination therapy. This multi-targeted approach can decrease the likelihood of drug resistance developing over the course of treatment [102]. For this to be successful, the correct ratio of drugs must arrive at the target site within the same timeframe; otherwise, toxicity issues may result. Therefore, nanoparticles have been developed to deliver multiple drugs at an optimised ratio.

Researchers have recently been combining covalent tyrosine kinase inhibitors (TKIs) with traditional anticancer drugs, such as cisplatin [103] and doxorubicin [99,104]. Morton et al. (2014) synthesised PEGylated liposomes to investigate the synergistic effects of covalent TKIs with cisplatin or doxorubicin, which were compared to the synergistic effects of first-generation (non-covalent) TKIs cisplatin or doxorubicin. The doxorubicin–afatinib combination was found to be the fastest at inducing apoptosis against BT-20 triple-negative breast cancer and A549 non-small-cell lung cancer cell lines in vitro, out of all the doxorubicin–TKI combinations. Furthermore, the cisplatin–afatinib combination produced the highest maximal amount of apoptosis (~20%) in A549 cells [104].

**Figure 11:**
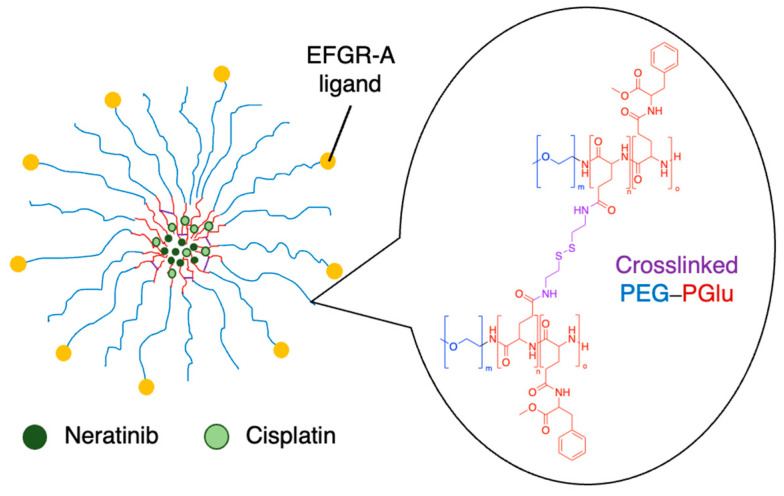
Co-loaded neratinib and cisplatin nanogels made from crosslinked PEG–PGlu [105].

The synergistic effects of cisplatin and TKIs were investigated against nasopharyngeal carcinoma. Afatinib was co-delivered with cisplatin in lipid–polymer hybrid nanoparticles made from PLGA, PEG and various lipids. They found that the anticancer effects of the co-delivered drugs were remarkably improved in cell viability, cell cycle, apoptosis and cell migration assays, as well as in a xenograft model [103]. In another study, polymeric nanogels made from PEG–PGlu block copolymers were modified with EFGR-A protein ligands and co-encapsulated cisplatin and the TKI neratinib (Figure 11) [105]. Part of the glutamic acid blocks were modified with hydrophobic groups, and another part was crosslinked via the carboxylic acid side chains, improving stability. Cisplatin coordinated with the carboxylate groups of the polymer, while neratinib interacted with the hydrophobic Phe domains that were installed on the polymer. The nanogels improved the activity of the drugs in EGFR(+) ovarian cancer xenografts compared to the free drugs [105].

The inconveniences of paclitaxel being a weekly intravenous administration and afatinib being an oral daily administration support the development of a drug delivery method for these drugs. It has been suggested that sequential application of anticancer drugs can lead to an enhanced effect [106]. The PLGA porous microspheres designed by Yang et al. (2019) could load both paclitaxel and afatinib-encapsulated solid-lipid nanoparticles. This enabled a two-phase release: an initial burst release of the paclitaxel followed by a sustained release of the afatinib [76].

## 3. Conclusions and Future Directions

Polymeric nanoparticles offer multiple benefits for the delivery of covalent drugs, in terms of solubility, permeability, lifetime, selectivity, controlled release and synergy with other drugs. These benefits should mitigate the concerns that have historically been expressed within some parts of the pharmaceutical industry about the potential toxicity and susceptibility to metabolism of covalent drugs as a general class. Indeed, the option of drug delivery may liberate medicinal chemists to focus more on potency, without making too many concessions towards the complicating factors of pharmacokinetic properties, thereby opening new possibilities for disease treatment in the future. With that long-term goal in mind, it is important to acknowledge that the majority of examples presented in this review represent early-stage research efforts; nevertheless, it is exciting to track the progress of some leading examples that have entered clinical trials [107,108,109].

## Figures and Tables

**Table 1:** Various nanoparticle systems for poorly soluble covalent drugs.

Entry	Drug	Nanoparticle Type	Significant Findings	Ref.
1	Orlistat	PBA-hyaluronic acid nanoparticles	97% encapsulation efficiency (EE); 19% drug loading capacity (LC)	[46]
2	Orlistat	Hyaluronic acid–lipid–polymer hybrid nanoparticles	90% EE; 6% drug LC	[47]
3	Orlistat	PLGA-PEG nanoparticles	72% EE; 7% drug LC	[48]
4	Orlistat	Polydopamine-coated hollow capsules	91% EE (using Nile Red as proxy drug)	[49]
5	Ibrutinib	Pluronic-stabilised nanosuspension	21-fold increase in solubility	[50]
6	Ibrutinib	Pluronic-stabilised PLGA nanoparticles	4-fold enhancement of oral bioavailability	[51]
7	Ibrutinib	Cyclodextrin chitosan nanoparticles	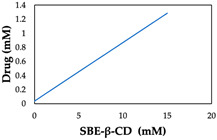 77% EE; 13% drug LC	[52]

**Table 2:** Nanoparticle systems designed to cross various biological membranes.

Entry	Drug	Nanoparticle Type	Biological Barrier	Ref.
1	Amoxicillin	Thiolated chitosan/PMLA nanoparticles	Stomach	[57]
2	5-Fluorouracil	Chitosan-pluronic nanogels	Skin	[58]
3	Cyclophosphamide	Polyalkylcyanoacrylate nanospheres	Eye	[55]
4	Afatinib	PLGA nanoparticles	Lung	[59]
5	Afatinib	PLGA nanoparticles	Lung	[60]
6	Carmustine	Solid lipid nanoparticles conjugated with lactoferrin	BBB	[61]
7	Saxagliptin	Chitosan nanoparticles with valine	BBB	[23]
8	Afatinib	Lipid–polymer nanoparticles with tight junction-modulating peptides	BBB	[62]

**Table 3:** Various nanoparticle systems that improve the lifetime of covalent drugs.

Entry	Drug	Nanoparticle Type	T_1/2_ (h) (Free Drug vs. NP Drug)	Ref.
1	Ibrutinib	Crosslinked human serum albumin	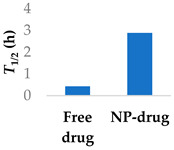	[71,72]
2	Ibrutinib	Lipid–polymer hybrid nanoparticles	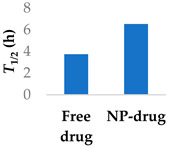	[73]
3	Afatinib	PEGylated liposomes	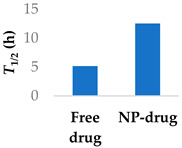	[74]
4	Afatinib	Tf modified redox-sensitive lipid–polymer hybrid nanoparticles	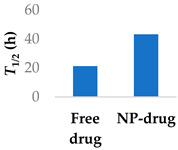	[75]
5	Afatinib	SLN in PLGA large porous particles	81 h (NP-drug)	[76]
6	Carmustine	PLGA-chitosan core-shell nanoparticles	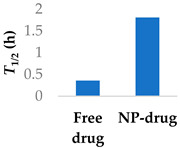	[77]

**Table 4:** Active targeting of covalent drugs with various nanoparticle systems.

Entry	Drug	Nanoparticle type	Targeting moiety	Significant findings	Ref.
1	Bortezomib	Crosslinked chitosan nanoparticles	CD38-targeting antibody	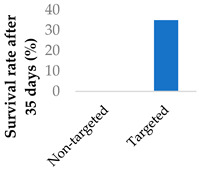	[82]
2	5-Fluorouracil	PEGylated liposomes	Folate	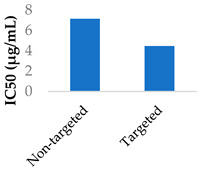	[83]
3	Mitomycin C	PEG-lipid-PLA-SPC hybrid nanoparticles	Folate	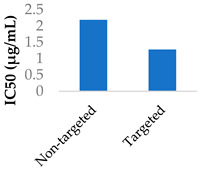	[84]
4	Afatinib	Lipid–polymer hybrid nanoparticles	Transferrin	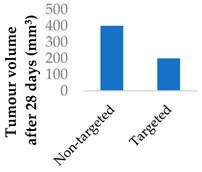	[75]
5	Decitabine	Lipid–polymer hybrid nanoparticles	Alendronate	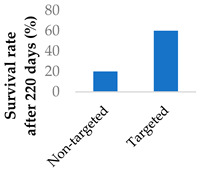	[85]
6	Mitomycin C	Terpolymer-lipid hybrid nanoparticles	Peptide iRGD	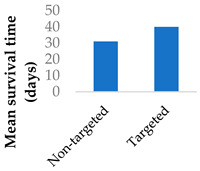	[86]
7	Sotorasib	Self-assembled hyaluronic acid-TPP nanoparticles	Hyaluronic acid	Significantly higher killing effect on mutant p53 cells vs. normal and non-mutant carcinoma cells	[87]

**Table 5:** Examples of how drug release can be controlled in nanoparticle systems.

Entry	Drug	Nanoparticle Type	Release Kinetics	Ref.
1	Mitomycin C	PLA-SPC nanoparticles *^a^*	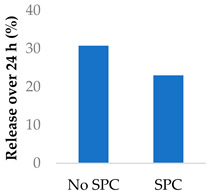	[93]
2	Mitomycin C	PLA-SPC nanoparticles	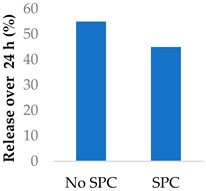	[94]
3	Mitomycin C	PEG-lipid-PLA-SPC hybrid nanoparticles	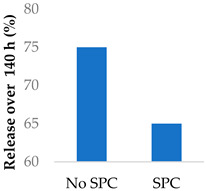	[95]
4	Mitomycin C	PEGylated liposomes	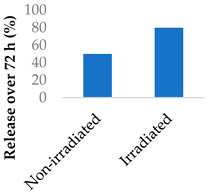	[96]
5	5-Fluorouracil	Magnetite nanographene oxide PCL nanoparticles	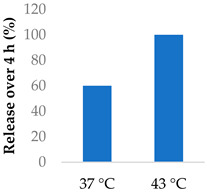	[97,98]
6	Afatinib	PEG-P(Asp(DBA)-co-Phe) polymeric nanovesicles	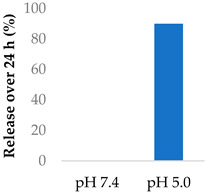	[99]
7	Mitomycin C	Crosslinked PVA-SA nanoparticles	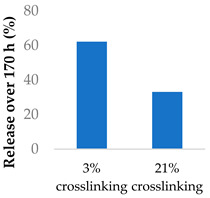	[100]
8	Ampicillin	PVA/chitosan nanofibers	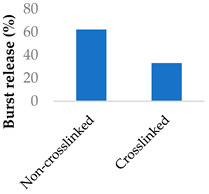	[101]

*^a^* SPC = soybean phosphatidylcholine; also see Figure 10.

## Data Availability

Not applicable.
